# Development and First-in-Human Translation of Hyperpolarized [1-^13^C]Alpha-Ketoglutarate MR Spectroscopy in the Brain

**DOI:** 10.3390/s26092753

**Published:** 2026-04-29

**Authors:** Yaewon Kim, Duy Dang, James Slater, Andrew Riselli, Donghyun Hong, Jeremy W. Gordon, Susan M. Chang, Yan Li, Javier E. Villanueva-Meyer, Adam W. Autry, Evelyn Escobar, Stacy Andosca, Hsin-Yu Chen, Chou T. Tan, Chris Suszczynski, Sri Maddali, Robert A. Bok, Daniel B. Vigneron

**Affiliations:** 1 Department of Radiology and Biomedical Imaging, University of California, San Francisco, CA 94158, USA; yaewon.kim@ucsf.edu (Y.K.); duy.dang@ucsf.edu (D.D.); jim.slater@ucsf.edu (J.S.); donghyun.hong@ucsf.edu (D.H.); jeremy.gordon@ucsf.edu (J.W.G.); yan.li@ucsf.edu (Y.L.); javier.villanueva-meyer@ucsf.edu (J.E.V.-M.); adam.autry@ucsf.edu (A.W.A.); hsin-yu.chen@ucsf.edu (H.-Y.C.); robert.bok@ucsf.edu (R.A.B.); 2 Department of Neurological Surgery, University of California, San Francisco, CA 94158, USA; susan.chang@ucsf.edu; 3 ISOTEC Stable Isotope Division, MilliporeSigma, Merck KGaA, Miamisburg, OH 45342, USA

**Keywords:** alpha-ketoglutarate, glutamate, hyperpolarized ^13^C MRI, IDH-mutants

## Abstract

Alpha-ketoglutarate (aKG) is a central intermediate of cerebral energy metabolism and a precursor for glutamate synthesis in the brain. Alterations in aKG metabolism occur in pathological contexts, including isocitrate dehydrogenase (IDH) mutant astrocytomas and oligodendrogliomas, in which mutant IDH converts aKG to the oncometabolite 2-hydroxyglutarate. Given its central role in brain metabolism, non-invasive interrogation of aKG-dependent metabolic flux is needed. Hyperpolarized (HP) ^13^C MR enables real-time visualization of metabolic conversion by transiently enhancing signal intensity by several orders of magnitude. Leveraging this approach, we report the first-in-human feasibility and safety study of HP [1-^13^C]aKG MR spectroscopy in the healthy brain (n = 3). A standard operating procedure (SOP) was developed for sterile [1-^13^C]aKG dose production, achieving reproducible polarization levels averaging 30.5 ± 2.2%. Following intravenous administration, time-resolved ^13^C spectra in healthy volunteers demonstrated the detection of HP aKG resonance and a measurable downstream glutamate signal, consistent across repeat acquisitions, with a delayed temporal profile relative to aKG observed in a representative dataset. Although performed in healthy volunteers, these results establish feasibility for HP [1-^13^C]aKG metabolic imaging to open a new window into normal and pathological brain cellular metabolism.

## 1. Introduction

Alpha-ketoglutarate (aKG) is a central intermediate of the tricarboxylic acid cycle and plays a pivotal role in cellular energy metabolism [1]. In the brain, aKG links mitochondrial oxidative metabolism to amino acid synthesis and neurotransmitter regulation, serving as a precursor for glutamate production [2]. Beyond its metabolic role, aKG also acts as a cofactor for aKG-dependent dioxygenases that regulate epigenetic processes and cellular differentiation [3,4], underscoring its broad biological importance.

Alterations in aKG metabolism occur in certain brain tumors as part of cellular metabolic reprogramming. Specifically, mutations in isocitrate dehydrogenase (IDH), frequently found in astrocytomas and oligodendrogliomas, disrupt the normal metabolic fate of aKG [5,6]. Whereas wild-type IDH catalyzes the conversion of isocitrate to aKG, mutant IDH instead reduces aKG to the oncometabolite 2-hydroxyglutarate (2-HG), which leads to metabolic and epigenetic dysregulation [7,8,9]. A tumor’s IDH mutation status has important prognostic and therapeutic implications and is associated with improved overall and progression-free survival, with reported five-year survival rates of 93% in IDH-mutant gliomas compared with 51% in IDH-wild-type gliomas [10,11]. In 2024, the U.S. Food and Drug Administration (FDA) granted traditional approval to the IDH inhibitor vorasidenib (VORANIGO, Servier Pharmaceuticals, LLC) for the treatment of IDH-mutant grade 2 astrocytoma and oligodendroglioma, further underscoring the clinical relevance of IDH-associated metabolism [12,13]. Given the central role of aKG in brain metabolism and its clinical relevance to IDH-mutant gliomas, non-invasive interrogation of aKG-dependent metabolic flux could provide valuable insights into both physiological and pathological states, including diagnosis and monitoring of the response to IDH-targeted therapy.

Hyperpolarized (HP) ^13^C magnetic resonance (MR) enables real-time visualization of metabolic conversion by increasing signal intensity by several orders of magnitude [14], thereby permitting direct assessment of substrate-to-product flux in vivo [15,16,17]. To date, HP [1-^13^C]pyruvate has established the clinical feasibility of metabolic imaging across 17 universities and over 2000 human studies [18], the development of a new HP ^13^C probe, [1-^13^C]aKG, would enable interrogation of a key metabolic branch point relevant to both normal brain function and IDH-associated pathology [19]. Preclinical studies have shown that HP [1-^13^C]aKG can detect conversion to glutamate and aberrant 2-HG production in IDH-mutant glioma models [20,21,22], as well as monitor response to IDH-targeted therapies [23,24]. Despite this strong biological rationale, translation of HP [1-^13^C]aKG to human studies requires reliable probe preparation, sterile clinical administration, regulatory FDA and IND approval, and optimization of acquisition strategies for the human brain.

In this project, we developed novel methods for the hyperpolarization of aKG and sterile dose production and then performed the first-in-human studies of HP [1-^13^C]aKG MR spectroscopy in healthy volunteers. We developed a standard operating procedure (SOP) for sterile administration and MR acquisition methods for the detection of aKG and its downstream metabolite glutamate in the healthy human brain. This demonstration of safe HP aKG administration and measurable metabolic conversion provides a translational foundation for future investigations of IDH-associated metabolism in patients with gliomas.

## 2. Materials and Methods

### 2.1. Hyperpolarized [1-^13^C]Alpha-Ketoglutarate (aKG) Preparation for Human Studies

Hyperpolarized (HP) [1-^13^C]aKG was prepared using GMP-grade [1-^13^C]aKG (ISOTEC, MilliporeSigma, Merck KGaA, Miamisburg, OH, USA) under FDA-IND-approved preparation procedures. Briefly, 1.30 g of [1-^13^C]aKG was dissolved in a mixture of ethanol (Sigma-Aldrich, St. Louis, MO, USA) and H_2_O (60:40 *v*/*v*), together with 15 mM of the electron paramagnetic agent (EPA) AH111501 (GE HealthCare, Waukesha, WI, USA). Ethanol was used as a co-solvent to facilitate dissolution of aKG and promote formation of a homogeneous glassy matrix during dynamic nuclear polarization (DNP), which is critical for uniform distribution of the EPA and efficient polarization buildup. Although aKG exhibits lower solubility in ethanol than in water, the selected solvent composition represented a practical balance among achieving high probe concentration for clinical dosing, maintaining preparation efficiency, and ensuring sufficient ethanol content for reliable glass formation required for DNP.

The solution was vortexed for approximately 1 h to ensure complete dissolution and homogeneity. When necessary, the [1-^13^C]aKG powder was gently ground using a mortar and pestle prior to mixing to facilitate solubilization. A total of 1.44 g of the resulting solution (5.6 M [1-^13^C]aKG and 15 mM EPA) was loaded into a cryovial, assembled into a fluid path, and polarized for 3 h using the clinical polarizer (SPINLab, GE Healthcare, Milwaukee, WI, USA). Polarization was performed at a microwave frequency of 139.87 GHz with a microwave power of 1 dB, using parameters previously optimized for HP [1-^13^C]pyruvate and found to be optimal for [1-^13^C]aKG. The polarization buildup followed a mono-exponential behavior with a time constant of 3306 ± 284 s (n = 5).

Following polarization, the frozen aKG sample was rapidly dissolved using 42 mL of preheated sterile water for injection (SWFI) and passed through a mechanical filter (pore size 13 μm) using a GE Fluid Handler system to remove EPA from the dissolution solution. The solution was subsequently neutralized with buffer solution (14.5 g of 333 mM Tris/EDTA/NaOH buffer combined with 17.35 g of SWFI), sterile-filtered through a 0.22 µm biosafety filter, and collected into a 60 mL Medrad (Bayer HealthCare LLC, Whippany, NJ, USA) syringe under pharmacist oversight. Within one minute after dissolution, approximately 43 mL of injectable HP [1-^13^C]aKG solution was produced. A detailed list of materials and quantities used in the preparation is provided in Table S1.

A 3 mL aliquot of the final product was retained for quality control (QC) testing, which was performed both prior to product release and after administration. Pre-release QC included visual inspection for residual EPA by comparison with a 5 µM reference standard (AH111501), pH assessment using pH strips, verification of final product volume, and sterile filter integrity testing. The intrinsic color of AH111501 enabled rapid qualitative estimation of residual EPA concentration, and comparison to the 5 µM reference standard ensured that residual EPA levels were below acceptable limits. Given the time-sensitive nature of HP probe administration, QC procedures were designed to minimize dissolution-to-injection time. Post-administration QC testing included pH measurement using a calibrated electrode, as well as endotoxin and sterility testing. All quality control parameters were evaluated against the release criteria summarized in Table S2.

### 2.2. Characterization of Hyperpolarized [1-^13^C]Alpha-Ketoglutarate (aKG) Solution

T_1_ relaxation times and liquid-state polarization levels of HP [1-^13^C]aKG were measured using a benchtop NMR spectrometer (Spinsolve 60, Magritek GmbH, Aachen, Germany) immediately following dissolution. The HP signal decay curve was acquired using 1° RF excitations every 5 s for 128 repetitions. The *T*_1_ for [1-^13^C]aKG was determined from an exponential fit to the dynamic HP signal intensities after correcting for the signal loss caused by RF pulses. For measurement of thermal equilibrium signal, samples were doped with 1% *v*/*v* Gd-DTPA (Magnevist^®^, Bayer, Whippany, NJ, USA) to shorten *T*_1_ relaxation time. The thermal equilibrium signal intensity was acquired using a 90° flip angle, 10 s repetition time, and 512 averages. Polarization was calculated by extrapolating the HP signal to the time of dissolution using the fitted *T*_1_ and comparing it to the corresponding thermally equilibrated signal, with corrections applied for differences in acquisition parameters.

Solution concentration and chemical composition were further characterized using high-field 400 MHz NMR spectroscopy (Bruker Avance III, Bruker, Rheinstetten, Germany). Quantitative ^13^C NMR measurements were performed using an external calibration curve generated from reference solutions of known carbon concentration to determine the concentration of the final [1-^13^C]aKG solution. Structural confirmation of [1-^13^C]aKG and identification of minor species were performed using ^1^H NMR and two-dimensional experiments (e.g., HMBC, HSQC, and COSY) as needed.

### 2.3. Preclinical Toxicology and Safety Assessment

Preclinical toxicology studies were conducted in male Sprague-Dawley rats (2–4 months old, 0.33–0.50 kg body weight) following intravenous administration of HP [1-^13^C]aKG injection product. For all animals, 3.0–3.5 mL of solution was administered via tail vein injection over 10–12 s. A total of seven animals were studied, including a saline control group (n = 3) and an HP [1-^13^C]aKG group (n = 4).

Animals were monitored before, during, and after injection for vital signs and clinical signs of toxicity and were followed for two weeks post-injection to assess survival and changes in body weight. Blood samples were collected at predefined time points (baseline, 20 min, and 2 weeks post-injection) for complete blood counts and liver–kidney function testing. At the end of the monitoring period, animals were euthanized for gross pathological examination of major organs.

### 2.4. Human Subject Hyperpolarized ^13^C MR Study Protocol

All imaging experiments were performed in healthy adult volunteers under an institutional review board-approved protocol, with written informed consent obtained from all participants prior to enrollment. A total of three healthy subjects participated in the study. Subjects had no known neurological disease or contraindications for MRI. HP [1-^13^C]aKG imaging was conducted to assess feasibility in the healthy volunteer brain.

**Figure 1:**
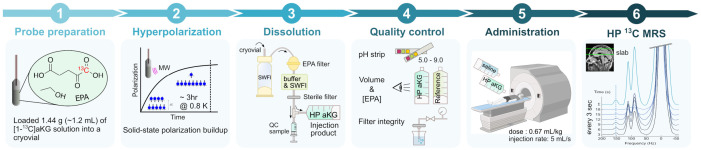
Overview of the clinical-research workflow of HP [1-^13^C]aKG production and HP ^13^C MRS.

A sterile dose of the HP [1-^13^C]aKG solution was administered intravenously at a dose of 0.67 mL/kg at an injection rate of 5 mL/s, followed by a 20 mL saline flush. MR data acquisition started 3 s after the completion of saline injection. Each subject received two injections separated by at least 15 min to evaluate the repeatability and stability of signal detection within the same imaging session. Subjects were continuously monitored throughout the imaging session in accordance with institutional safety guidelines for clinical MRI and the approved IND protocol. No adverse events were observed during or after administration of the HP [1-^13^C]aKG probe, and all MRI sessions were completed successfully. The overall workflow for probe preparation, dissolution, quality control, and in vivo spectroscopy is summarized in Figure 1.

### 2.5. MR Data Acquisition

All MR experiments were performed on a 3T clinical MRI system (SIGNA Premier, GE Healthcare) using a dual-tuned transmit-receive head coil (8-ch ^1^H/ 24-ch ^13^C). Dynamic slab-based ^13^C MR spectroscopy (MRS) was performed with a slab thickness of 6 cm. Dynamic ^13^C spectra were acquired every 3 s for a total duration of 60 s. Higher-order B_0_ shimming was performed prior to ^13^C MRS acquisition to improve magnetic field homogeneity.

A 10.2 ms multiband spectral-spatial RF excitation pulse was designed to apply a small flip angle (2°) to the [1-^13^C]aKG resonance, while a higher flip angle (40°) was applied to a metabolite band centered approximately 262 Hz downfield from the C_1_-aKG resonance with a passband of ±148.5 Hz that encompassed glutamate and 2-HG resonances. Chemical shift induced slice displacement was corrected during pulse design. The excitation bandwidth encompassed the expected resonance of 2-HG to enable future studies in patients with IDH-mutant gliomas. Acquisition parameters were kept consistent across subjects and repeated injections to assess measurement repeatability within the imaging session. In one subject, a higher flip angle of 60° was applied in the first injection, whereas 40° was used in the second injection. The selected flip angles were chosen empirically based on prior preclinical studies to balance preservation of substrate magnetization and detection of low-SNR metabolic products in this initial feasibility study. For anatomical reference, T_2_-weighted FLAIR images were acquired.

### 2.6. Data Processing and Analysis

All ^13^C MR data were processed using MATLAB 2023b (MathWorks, Natick, MA, USA). Raw multi-channel free induction decay (FID) data were first pre-whitened to decorrelate receiver noise across channels [25]. The pre-whitened FID data were then truncated to retain the first half of the acquired time-domain data points to reduce late-time noise, followed by Fourier transformation with exponential apodization corresponding to a 4 Hz line broadening and zero-filling by a factor of four.

Denoising was performed using tensor decomposition (TD)-based regularization [26] applied to the complex-valued 3D dataset spanning frequency, dynamic time frames, and receiver channels. A tensor rank of (10, 20, 10) was used for the frequency, temporal, and channel dimensions, respectively, with ranks selected empirically to preserve spectral features and avoid artificial temporal correlations. A comparison of raw and denoised data is provided in the Supplementary Information (Figure S1) for reference.

Following denoising, receiver channel signals were combined using a Roemer optimal combination approach [27], with coil sensitivity weighting derived from the integrated peak area of the HP [1-^13^C]aKG signal. Only receiver channels within the prescribed 6 cm slab were included in the coil combination to maximize SNR and minimize signal dilution by noise. This corresponded to approximately 6–15 of the 24 available ^13^C channels, depending on slab positioning. All spectra were referenced to the [1-^13^C]aKG resonance at 172.6 ppm.

To enhance SNR and improve visualization of downstream metabolites, time-integrated spectra were generated by summing complex-valued spectra across the first six dynamic time frames to form area-under-the-curve (AUC) spectra. This approach enhances coherent signal accumulation while suppressing incoherent noise contributions. Zero- and first-order phase correction and spline-based baseline correction were subsequently performed using TopSpin 4.5.0 (Bruker BioSpin, Rheinstetten, Germany) for spectral visualization.

## 3. Results

### 3.1. Characterization of HP [1-^13^C]aKG Solution

**Figure 2:**
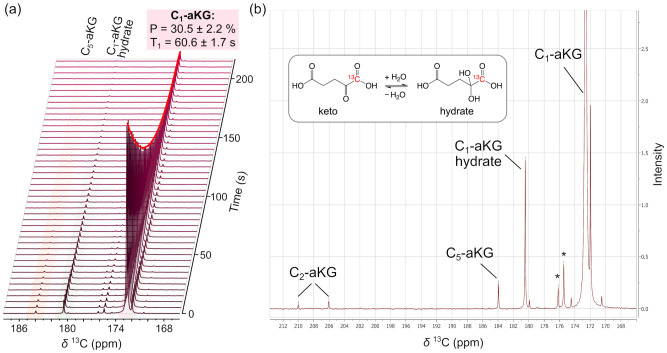
Time-resolved ^13^C NMR spectra of HP [1-^13^C]aKG acquired at 1.4T with a temporal resolution of 5 s, demonstrating exponential signal decay used for *T*_1_ estimation. (**b**) Expanded view of the first time frame from (**a**), showing the ^13^C resonances of HP [1-^13^C]aKG corresponding to the keto and hydrated forms, along with minor esterified species (*).

Hyperpolarized [1-^13^C]alpha-ketoglutarate (aKG) was polarized using the GE SPINlab polarizer (GE Healthcare, Milwaukee, WI, USA) and prepared for intravenous administration in human subjects. Following dissolution and neutralization, the final solution pH was pH 8.1 ± 0.2, within the QC release criteria (pH 5.0–9.0). The HP [1-^13^C]aKG solution achieved high liquid-state polarization levels suitable for in vivo MR spectroscopy. The mean HP [1-^13^C]aKG concentration and polarization level were 88.6 ± 3.7 mM and 30.5 ± 2.2%, respectively (n = 6, corresponding to the six injections performed across three subjects). The longitudinal relaxation time (*T*_1_) of the C_1_-aKG resonance was measured as 63.1 ± 1.8 s at 3T and 60.6 ± 1.7 s at 1.4 T (Figure 2a).

As shown in Figure 2b, the representative ^13^C NMR spectrum of the [1-^13^C]aKG probe demonstrated distinct resonances corresponding to C_1_-aKG (172.6 ppm; keto), C_1_-aKG hydrate (180.5 ppm), C_5_-aKG (183.9 ppm), and C_2_-aKG (206.0 and 210.0 ppm), the latter appearing as a doublet due to ^13^C-^13^C coupling. Thermal ^13^C NMR analysis confirmed that [1-^13^C]aKG predominantly existed in the keto form, with 5.9 ± 0.4% present as the hydrated species under equilibrium conditions at pH 8.

Minor esterification products arising from interaction of [1-^13^C]aKG with ethanol during formulation were observed at 175.4 and 176.2 ppm, accounting for less than 1% of the keto-aKG concentration. These resonances are consistent with low levels of monoethyl and diethyl ester derivatives of [1-^13^C]aKG. Prior reports indicate that C_1_-esterified aKG is more labile than the regioisomeric C_5_-ester, which may contribute to the relative predominance of more stable ester species under the formulation conditions [28].

Residual ethanol content in the final injectable formulation was approximately 74 mM (0.43% *v*/*v*), corresponding to ~0.14 g ethanol for a 60 kg subject (~2.3 mg/kg). This level of ethanol exposure is substantially lower than concentrations reported in FDA-approved intravenous formulations and falls within ranges generally considered low risk for clinical administration [29].

### 3.2. Preclinical Toxicology and Safety Evaluation

Preclinical toxicology studies conducted for the Investigational New Drug (IND) application (approved by the FDA and our IRB) demonstrated no evidence of acute or chronic toxicity following intravenous administration of [1-^13^C]aKG. No adverse effects were observed in either the saline control group or the HP [1-^13^C]aKG group. All injections were well tolerated, with no significant changes in physiological parameters, including heart rate, respiratory rate, or oxygen saturation (Table S3A–C).

Animals also exhibited no significant changes in body weight, complete blood counts, or liver–kidney function parameters, and no mortality was observed during the two-week post-injection monitoring period (Table S3D–F). Laboratory analyses revealed no significant deviations from baseline values, saline controls, or established reference ranges at either acute or chronic time points. No behavioral changes were observed, and gross pathological examination performed two weeks after injection revealed no abnormalities in the major organs.

### 3.3. Hyperpolarized ^13^C MRS in the Human Brain

**Figure 3:**
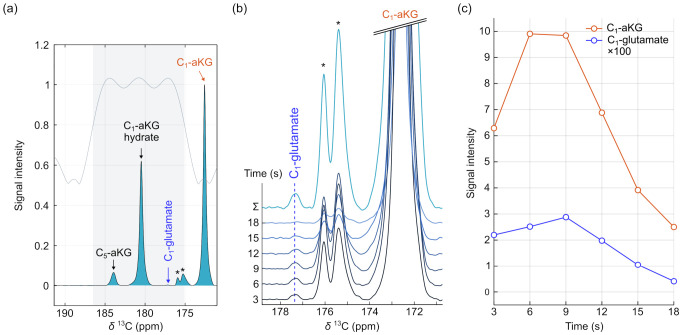
In vivo hyperpolarized [1-^13^C]aKG MR spectroscopy in a healthy volunteer. (**a**) Summed ^13^C spectrum from the first six dynamic time frames acquired in the brain. (**b**) Dynamic spectra showing the C_1_-glutamate resonance following injection, along with C_1_-aKG and minor esterified species (*). (**c**) Time courses of C_1_-aKG (orange line) and C_1_-glutamate (blue line) signal integrals.

Hyperpolarized ^13^C MR spectroscopy was successfully performed in healthy volunteers following intravenous administration of HP [1-^13^C]aKG. Time-integrated spectra generated from the first six dynamic frames demonstrated detection of the HP [1-^13^C]aKG resonance within the prescribed brain slab (Figure 3a). As designed, the spectral-spatial RF excitation applied a low flip angle to the C_1_-aKG and a higher flip angle to downstream metabolites including glutamate (see gray overlay in Figure 3a), yet C_1_-aKG remained the dominant resonance, reflecting effective substrate delivery and preserved polarization.

The spectral region corresponding to C_1_-glutamate was examined dynamically (Figure 3b). The HP C_1_-glutamate was detectable in individual dynamic time frames, and signal accumulation across early time frames further enhanced visualization of this resonance. Quantitative analysis of the dynamic time courses revealed distinct temporal profiles for substrate and product signals (Figure 3c). The C_1_-aKG signal peaked shortly after injection and decayed in a manner consistent with vascular delivery and *T*_1_ relaxation of the HP substrate. In contrast, the C_1_-glutamate signal exhibited a delayed rise relative to C_1_-aKG, consistent with downstream enzymatic conversion.

**Figure 4:**
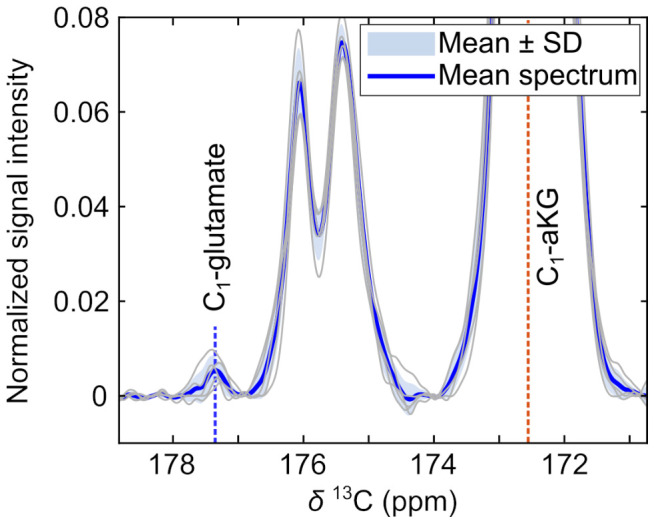
Detection of C_1_-glutamate across healthy volunteers. Mean summed spectrum (blue line) across five datasets with individual spectral traces displayed in gray (mean ± SD shading). Spectra were frequency-aligned to the C_1_-aKG peak prior to averaging. The dashed line indicates the expected C_1_-glutamate resonance.

To evaluate consistency across datasets, the remaining five datasets were analyzed separately (Figure 4). After frequency alignment to the C_1_-aKG resonance, the C_1_-glutamate peak was observed in all datasets within −4.9 ± 1.6 Hz of its expected position (157.4 Hz relative to C_1_-aKG at 3T), indicating consistent spectral localization. In time-integrated spectra, C_1_-glutamate SNR ranged from 1.2 to 6.2 across datasets. To account for variability in substrate polarization and overall signal amplitude, glutamate SNR was normalized to the corresponding C_1_-aKG SNR. Repeat acquisitions within individual volunteers showed glutamate-to-aKG ratios that were generally comparable (e.g., 0.0065 vs. 0.0070 and 0.0028 vs. 0.0037), although some variability was observed. Glutamate-to-aKG ratios and corresponding glutamate and aKG SNR values for all datasets are provided in Table S4.

## 4. Discussion

This first-in-human study demonstrated the feasibility and safety of HP [1-^13^C]aKG MR metabolic spectroscopy in the healthy human brain. The specialized SOP workflow enabled sterile probe production with reproducible polarization levels averaging approximately 30%, permitting in vivo detection of aKG and its downstream metabolite glutamate. Because this study was performed exclusively in healthy volunteers, no 2-HG was observed, consistent with intact wild-type IDH activity. Notably, aKG has a well-established history of intravenous administration in humans for multiple clinical indications, including stroke treatment [30], perioperative metabolic support [31], cardioplegia in cardiac surgery [32], and nutritional supplementation [33]. This prior clinical experience further supports the favorable safety profile observed in the present study.

The glutamate signal detected in the normal brain demonstrated consistent spectral localization across subjects in time-integrated spectra. A delayed temporal profile relative to substrate delivery was observed in the representative dataset (Figure 3). Under the current experimental conditions, glutamate signals were variably detectable in dynamic spectra and more reliably visualized in time-integrated spectra, indicating that the current setup operates near the practical sensitivity limit. Accordingly, the present study focused on robust qualitative and semi-quantitative characterization of aKG-to-glutamate conversion, supported by prior work demonstrating the clinical utility of model-free approaches [34]. The observed variability in metabolite SNR and glutamate-to-aKG ratios across datasets likely reflects a combination of human brain heterogeneity, individual variation, differences in substrate delivery, and measurement variability, in which small fluctuations in signal amplitude and noise can lead to larger relative differences. Future studies with larger cohorts and improved spatial localization and SNR are needed to enable more reliable quantitative characterization of aKG metabolism.

The observed glutamate amplitude likely reflects physiological constraints, including limited blood–brain barrier (BBB) permeability of aKG [35,36] and tightly regulated glutamate homeostasis [1], rather than a fundamental technical limitation of the HP [1-^13^C]aKG platform. Transport of aKG across the intact BBB is restricted by its charged nature and the limited presence of dicarboxylic transporters at the endothelial interface [37,38]. In contrast, substrates such as pyruvate benefit from efficient transport via monocarboxylate transporters [39,40], resulting in higher brain delivery and signal intensity in hyperpolarized studies. While aKG delivery to the normal brain is likely limited by the intact BBB, delivery to tumor tissue may differ. Prior preclinical studies have demonstrated that HP [1-^13^C]aKG is delivered to the brain and can access tumor regions in IDH-mutant glioma models [21,23]. Although IDH-mutant gliomas often exhibit relatively intact BBB characteristics compared to higher-grade tumors, regional heterogeneity in vascular permeability may still permit substrate delivery. Therefore, HP [1-^13^C]aKG may still be well-suited for tumor applications, enabling direct interrogation of IDH-associated metabolism, including aberrant production of 2-hydroxyglutarate (2-HG).

HP ^13^C studies using esterified, cell-permeable derivatives of aKG have demonstrated improved intracellular delivery and enhanced downstream metabolite detection in preclinical settings [41]. However, translation of such chemically modified probes to human studies presents substantial challenges related to large-scale synthesis, stability under polarization conditions, and compliance with GMP manufacturing and regulatory requirements. In contrast, the present study establishes clinical feasibility using GMP-grade [1-^13^C]aKG suitable for human administration. Demonstration of measurable product signal under these constraints confirms practical detectability within a clinically translatable framework.

Having demonstrated the feasibility of probing cerebral aKG metabolism in the normal human brain, the broader objective of HP [1-^13^C]aKG MRS is detecting 2-HG in patients with IDH-mutant gliomas. Compared with proton (^1^H) MRS for 2-HG detection [42,43,44], HP ^13^C imaging offers the potential for dynamic assessment of substrate-to-product conversion within a short acquisition window. In contrast to PET [45], which involves ionizing radiation, HP ^13^C imaging is well-suited for repeated longitudinal examinations, enabling monitoring and therapeutic response assessment without cumulative radiation exposure [21].

Several technical refinements may further enhance clinical translation. First, the relatively short *T*_1_ of C_1_-aKG constrains the effective imaging window. Compared with HP [1-^13^C]pyruvate, C_1_-aKG exhibits a shorter *T*_1_ (approximately 10 s shorter under similar conditions), in part due to dipolar interactions between the carbonyl ^13^C nucleus and surrounding ^1^H nuclei in aqueous solution. Prior human HP ^13^C studies using D_2_O as a dissolution solvent have shown prolonged C_1_-pyruvate *T*_1_ through reduced heteronuclear dipolar relaxation, while also demonstrating safety and feasibility [46,47]. These findings suggest a practical strategy to readily increase available polarization and improve downstream SNR.

Second, improved spatial encoding will be important for patient studies. The present study employed slab-based spectroscopy to establish feasibility; however, this approach inherently limits spatial specificity and may include substantial vascular signal that cannot be readily separated from parenchymal metabolism. Vascular contributions are expected to be more pronounced for substrate signals, whereas downstream metabolites, such as glutamate, are less affected by intracellular conversion. Spatially resolved spectroscopic approaches, including chemical shift imaging or accelerated spectroscopic imaging techniques, would enable improved localization and separation of vascular versus parenchymal signals. Such refinements may be particularly relevant in gliomas, where metabolic alterations can be spatially heterogeneous.

Third, optimization of spectral specificity remains essential for reliable 2-HG detection at 3T. Spectral overlap between 2-HG and the C_5_ resonance of aKG presents a challenge for direct separation and quantification using [1-^13^C]aKG. Selective labeling strategies that suppress the C_5_-aKG resonance through substitution with ^12^C at C_5_ position, such as [1-^13^C,5-^12^C]aKG [41], reduce this overlap and enhance spectral discrimination of potential 2-HG signals. Preclinical studies have demonstrated improved 2-HG detection using this selectively labeled probe in IDH-mutant models [23,36,41]. Building upon these findings, clinical implementation of HP [1-^13^C,5-^12^C]aKG may enable more specific interrogation of IDH-associated metabolism in patients with gliomas and potentially other cancers exhibiting IDH mutations.

The clinical relevance of HP aKG imaging is supported by the growing use of IDH inhibitors for management of glioma patients. As targeted therapies expand, non-invasive biomarkers capable of assessing pathway-specific metabolic response are increasingly needed. Given its favorable safety profile and potential for repeated administration, HP aKG imaging may serve as a complementary tool for longitudinal metabolic assessment in neuro-oncology.

## 5. Conclusions

In this study, we demonstrated the first-in-human feasibility and safety of hyperpolarized [1-^13^C]aKG MR metabolic spectroscopy in the brain, with successful detection of aKG and measurable downstream glutamate signal in healthy volunteers. These findings establish the technical and clinical feasibility of hyperpolarized [1-^13^C]aKG MRS and provide a foundation for translational studies of IDH-associated metabolism in patients with gliomas. Ongoing optimization of hyperpolarized signal, spatial encoding, and spectral specificity, together with studies in larger cohorts, will further enhance this approach’s utility and enable more comprehensive analysis for non-invasive metabolic assessment and longitudinal monitoring in neuro-oncology.

## Data Availability

Data supporting the findings of this study are available from the corresponding author upon reasonable request.

## Supplementary Materials

The following supporting information can be downloaded at: https://www.mdpi.com/article/10.3390/s26092753/s1, Table S1: Composition of the Pharmacy kit (fluid path) for [1-^13^C]alpha-ketoglutarate (aKG) preparation; Table S2: Release criteria for [1-^13^C]alpha-ketoglutarate (aKG) injection product; Figure S1: Comparison of raw and Tensor decomposition (TD)-denoised in vivo spectra. Table S3: Physiological parameters measured before and after intravenous administration of HP [1-^13^C]aKG in preclinical toxicology studies. Table S4: Glutamate and aKG SNR values and glutamate-to-aKG ratios from time-integrated spectra across datasets.

## Abbreviations

The following abbreviations are used in this manuscript:

HP	Hyperpolarized
aKG	Alpha-ketoglutarate
SOP	Standard operating procedure
