# Supplementary material for: Development and First-in-Human Translation of Hyperpolarized [1-13C]Alpha-Ketoglutarate MR Spectroscopy in the Brain
Source: Sensors (Basel). 2026 Apr 29;26(9):2753. doi: 10.3390/s26092753 (PMC13165773; doi:10.3390/s26092753)
Supplement: Supplementary file 1 [file sensors-26-02753-s001.zip › sensors-4222105-supplementary.pdf]

Supporting Information for "*Development and First-in-Human Translation of Hyperpolarized [1-^{13}C]Alpha-Ketoglutarate MR Spectroscopy in the Brain*"

Yaewon Kim[1], Duy Dang[1], James Slater[1], Andrew Riselli[1], Donghyun Hong[1], Jeremy W. Gordon[1], Susan M. Chang[2], Yan Li[1], Javier E. Villanueva-Meyer[1,2], Adam W. Autry[1], Evelyn Escobar[1], Stacy Andosca[1], Hsin-Yu Chen[1], Chou T. Tan[3], Chris Suszczynski[3], Sri Maddali[3], Robert A. Bok[1], Daniel B. Vigneron[1,2]

[1]Department of Radiology and Biomedical Imaging, University of California, San Francisco, CA, USA.

[2]Department of Neurological Surgery, University of California, San Francisco, CA, USA.

[3]ISOTEC Stable Isotope Division, MilliporeSigma, Merck KGaA, Miamisburg, OH, USA

* Corresponding author:
Daniel B. Vigneron (dan.vigneron@ucsf.edu)

Table S1. Composition of the Pharmacy kit (fluid path) for [1-^{13}C]alpha-ketoglutarate (aKG) preparation

Fluid path component	Quantity per Batch	Function
2-Ketoglutaric acid-1-^{13}C	0.896 g (± 2%)	Active ingredient
EtOH and Sterile H_2O mixture (60:40 v/v)	0.515 g (± 2%)	Solvent
AH111501 (EPA)	0.0282 g (± 2%)	Electron Paramagnetic Agent
Sterile H_2O dissolution syringe (Part A)	42.00 g (± 0.05 g)	Solvent
Sterile H_2O in receiver bag (Part B)	17.35 g (± 0.05 g)	Solvent
333 mM Tris buffer (containing 1 mM EDTA, 600 mM NaOH)	14.50 g (± 0.05 g)	Neutralization

Table S2. Release criteria for [1-^{13}C]alpha-ketoglutarate (aKG) injection product

Test	Analytical Procedure	Acceptance Criteria
Residual AH111501 concentration	Visual inspection	< 5 µM
pH	pH strip	5.0 – 9.0
Drug product volume	Visual inspection	>38 mL
Filter Integrity	Bubble point test	Manufacturer specification
^{13}C Nuclear Polarization	Solid state NMR	> 100x increase

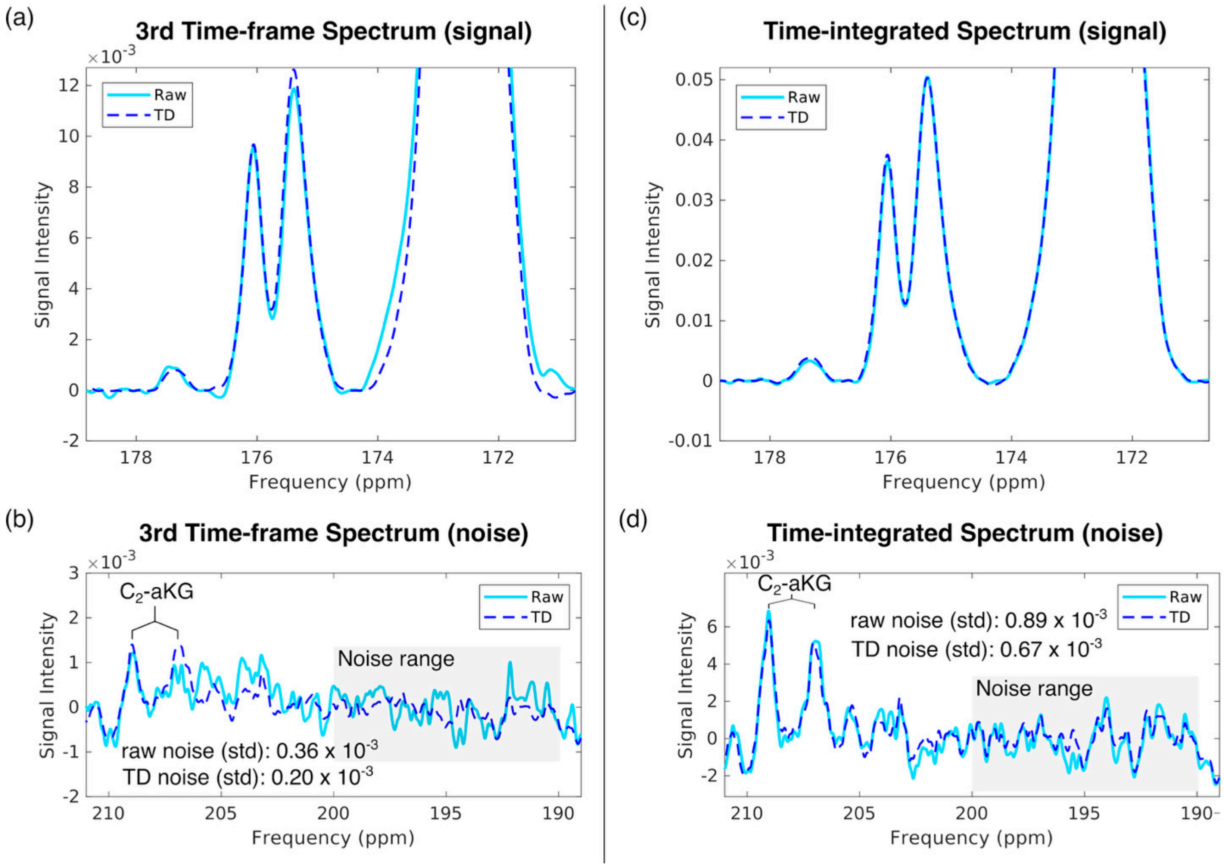

Figure S1. Comparison of raw and Tensor decomposition (TD)-denoised in vivo spectra. (a-b) Raw and TD-denoised spectra from the 3rd time frame of the Vol-1 scan, showing the (a) signal region and (b) noise region. In the noise spectrum, hyperpolarized C_2-aKG signals (doublet) are visible. The standard deviation of the noise region (indicated in gray) is reported in the panel. (c-d) Raw and TD-denoised spectra from time-integrated (frames 1-6) of the Vol-1 scan, showing the (c) signal and (d) noise region.

Table S3. Physiological parameters measured before and after intravenous administration of HP [1-^{13}C]aKG in preclinical toxicology studies

Table S3A. Heart rate (bpm)

Group	Injection	Baseline	5 min	10 min	15 min	20 min
I	Saline control	273 ± 16	279 ± 14	277 ± 10	280 ± 9	282 ± 21
II	HP aKG	352 ± 21	369 ± 12	354 ± 16	346 ± 16	347 ± 13

Table S3B. Breath rate (bpm)

Group	Injection	Baseline	5 min	10 min	15 min	20 min
I	Saline control	39 ± 7	38 ± 5	39 ± 6	41 ± 8	41 ± 8
II	HP aKG	57 ± 6	60 ± 9	57 ± 11	50 ± 6	52 ± 5

Table S3C. Oxygen saturation (%)

Group	Injection	Baseline	5 min	10 min	15 min	20 min
I	Saline control	99.5 ± 0.2	99.5 ± 0.1	99.5 ± 0.1	99.4 ± 0.1	99.5 ± 0.2
II	HP aKG	99.5 ± 0.4	99.6 ± 0.2	99.6 ± 0.4	99.6 ± 0.3	99.5 ± 0.4

Table S3D. Body weights 2 weeks post-injection (kg)

Group	Injection	SD1	SD3	SD5	SD8	SD10	SD14
I	Saline control	0.47	0.48	0.48	0.48	0.49	0.50
II	HP aKG	0.33	0.34	0.35	0.35	0.36	0.37

SD: study day

Table S3E. Complete Blood Count (CBC) at baseline, 20 minutes and 2 weeks post-injection

Group	Group I: Saline control (n = 3)			Group II: HP aKG (n = 4)			Normal Reference range
Time point	baseline	20 mins	2 weeks	baseline	20 mins	2 weeks	
**WBC**	12.7±1.5	9.2±3.1	11.7±2	11.5±4.4	10.5±4.9	11.8±4.4	6.0 - 18.0
**Neut%**	19±4.6	21.5±6.8	20.4±3.2	25.9±8.8	27.2±9.4	22.9±2.5	10.0-30.0
**Lymp%**	75.4±5.8	72.8±6.9	74.1±4.7	66.3±8.6	63.9±13.1	69.5±4.1	65.0-85.0
**Mono%**	4.3±0.9	5.3±0.8	4.5±0.7	6.9±3.2	7.8±6.0	5.3±1.8	0.0-5.0
**Eos%**	0.8±0.2	0.4±0.4	0.7±0.8	0.8±0.3	1.0±0.5	1.8±0.8	0.0-6.0
**Baso%**	0.5±0.2	0.1±0.1	0.7±0.3	0.1±0.0	0.1±0.1	0.6±0.4	0.0-1.0
**HGB**	15.4±0.9	14.5±0.4	14.6±1.4	15.3±0.8	13.6±0.4	15.8±0.8	11.0-19.2
**HCT**	46.1±4	42.7±0.5	44.2±0.9	45.8±2.2	40.8±1.2	46.8±3.2	36.0-54.0
**PLT**	971±161	1125 ± 430	1364 ± 299	1199±109	1145±130	897±207	500 - 1300

Abbreviations: WBC, white blood cells; Neut, neutrophils; Lymp, lymphocytes; Mono, monocytes; Eos, eosinophils; Baso, basophils; HGB, hemoglobin; HCT, hematocrit ; PLT, platelet count

Table S3F. Liver-kidney panel at baseline, 20 minutes and 2 weeks post-injection

Group	Group I: Saline control (n = 3)			Group II: HP aKG (n = 4)			Normal Reference range
Time point	baseline	20 mins	2 weeks	baseline	20 mins	2 weeks	
**ALT**	28.5±4.1	27.1±3.9	29.5±2.8	32.2±7.6	29.1±7.2	36.7±5.7	12 - 67
**AST**	59.6±15.9	64.4±29.4	68±7.7	88.6±9.3	63.6±17.9	93.4±26.1	14 - 113
**Albumin**	4±0.1	3.9±0.1	3.9±0.6	4.0±0.0	3.6±0.1	3.9±0.2	2.7 - 4.6
**Alk Phos**	106±1.2	106.1±4	107.1±12	244.6±97.2	224.1±93.3	207.5±65.0	21-367
**BUN**	14.5±1.1	19.7±3.3	16.7±4.6	17.5±2.4	16.8±1.2	18.2±2.0	10 - 25
**Creatine**	0.23±0.02	0.25±0.03	0.29±0.05	0.26±0.06	0.25±0.03	0.21±0.06	0.8 - 1.8
**Total Bilirubin**	0.066 ± 0.005	0.062 ± 0.033	0.126	0.028± 0.011	0.027± 0.018	0.070± 0.067	0.0 - 0.7
**Total Protein**	5.7±0.5	5.5±0.3	5.7±0.4	5.6±0.6	5.1±0.3	5.7±0.5	5.3 - 7.5

Abbreviations: ALT, alanine aminotransferase; AST, aspartate aminotransferase; Alk Phos, alkaline phosphatase; BUN, blood urea nitrogen

Table S4. Glutamate and aKG SNR values and glutamate-to-aKG ratios from time-integrated spectra across datasets.

Subject	Injection	Flip angle (°) for C_1-Glutamate	C_1-Glutamate AUC SNR	C_1-aKG AUC SNR	C_1-Glu/ C_1-aKG Ratio
Vol 1	Inj 1	60	6.5	1637	0.0039
Vol 1	Inj 2	40	6.2	635	0.0098
Vol 2	Inj 1	40	2.8	413	0.0065
Vol 2	Inj 2	40	3.1	443	0.0070
Vol 3	Inj 1	40	1.2	428	0.0028
Vol 3	Inj 2	40	1.8	478	0.0037
